# Phytochemicals and Functional Properties of Pitaya Juice Powders

**DOI:** 10.3390/plants13213040

**Published:** 2024-10-30

**Authors:** Mayra Denise Herrera, Jorge A. Zegbe, Luis Roberto Reveles-Torres

**Affiliations:** 1 Campo Experimental Zacatecas, Instituto Nacional de Investigaciones Forestales, Agrícolas y Pecuarias, Km 24.5 Carretera Zacatecas-Fresnillo, Calera de Víctor Rosales, Zacatecas CP 9500, Mexico; reveles.roberto@inifap.gob.mx; 2 Campo Experimental Pabellón, Instituto Nacional de Investigaciones Forestales, Agrícolas y Pecuarias, Km 32.5 Carretera Aguascalientes-Zacatecas, Pabellón de Arteaga, Aguascalientes CP 20668, Mexico

**Keywords:** *Stenocereus queretaroensis*, non-digestible carbohydrates, secondary metabolites antioxidant activity, pancreatic lipase inhibition

## Abstract

Background: Crassulacean acid metabolism plants, such as *Stenocereus* spp., are climate warming-resilient crops used as food and for by-products elaboration in arid and semi-arid agroecosystems. A few studies on secondary metabolites have been conducted in pitayo fruit (PF), but there are no reports of these compounds in juice powders (JP) with (JPS) or without seeds (JPWS). This study was devoted to characterizing the juice powders (JPS and JPWS) of five pitayas with different flesh colors with regard to some phytochemical and functional attributes. Methods: The study was conducted with a completely random design with factorial arrangement in treatments (PF × JP). Results: Differences among pitayas were related to peel and flesh color attributes. Except for soluble dietary fiber, the remainder of the non-digestible carbohydrates were greater in JPS than in JPWS of all pitayas. Phenols and flavonoids were found to be the highest in the JPWS of all pitayas, whereas total saponins were the highest in JPS of the ‘Pink’ pitaya. The JPWS of the ‘Yellow’ and ‘Reddish-Purple’ pitayas had the highest content of betaxanthins and indicaxanthins, respectively. Antioxidant capacity was the highest in JPS of ‘Reddish-Purple’ and ‘Pink’ pitayas. Conclusions: Except for some phenolic compounds, the study suggests that pitayas’ JPS would benefit human health when freshly consumed or as elaborated by-products.

## 1. Introduction

Plants exhibiting crassulacean acid metabolism (CAM) such as agave, *Aloe* spp., dragon fruit (*Hylocereus undatus*) pineapple (*Ananas comosus*), *Opuntia* spp., and *Vanilla* spp., are climate-resilient crops [1]. These crops have more opportunities to be grown when facing global warming than C3 and C4 crops because they are cultivated in arid and semi-arid agroecosystems. Another CAM ancient crop is the columnar cactus *Stenocereus* spp. [2]. Among the Cacti family, *Stenocereus* is a genus within the Caryophyllales that comprises ∼24 species of cacti. The main characteristic of this genus is the production of highly desired edible fruits, generically called “pitayas” [3]. The cultivated species of this genus are *S. thurberi*, *S. griseus*, *S. stellatus*, *S. fricci*, and *S. queretaroensis*, the latter being the most used due to the availability of genotypes and their productivity [4], but yet, the least studied. The pitayas harvested from this species are covered by a soft exocarp with thorny areoles which are detached in their phenological maturity during the spring. They have very small pyriform seeds, and its mesocarp is juicy and soft and ranges from blends of white and purple, although depending on the variety, it can also have colors ranging from pink to yellow these have acquired commercial value as exotic fruits worldwide [3,5]. However, as Soto-Castro et al. [6], mentioned in 2018, the *S. queretaroensis* fruits have not been studied enough; of late, the most common research dedicated not only to this species, but to pitayas of the genus is regarding the concentration of pigments, since the food, cosmetic, textile, and pharmaceutical industries are increasingly searching for natural pigments [7]. Betalains, a group of water-soluble pigments of a group of secondary metabolites in the Caryophyllales order are the most studied [8,9]. Nonetheless, plant pigments are a wide variety of compounds that can be grouped into green chlorophylls, yellow to orange carotenoids, red to blue anthocyanins, and red and yellow betalains, all of which have been studied for their functional properties [10]. Pitayas have been reported as efficient radical scavengers, however, only *S. pruinosus* [11], *S. stellatus* [12], and *S. thurberi* [13] were previously evaluated as ABTH and/or DPPH scavengers. Cervantes-Arista et al. [14] and García-Cruz et al. [15] related the *Stenocereus* fruits’ antioxidant capacity with the total phenol, flavonoid, and betalain content. Unlike prickly pear [16,17] and other fruits of the *Stenocereus* species [18,19], physical–chemical attributes of *S. queretaroensis* fruits have received little attention [20,21]. To this respect, Pimienta-Barrios and Tomas [21] pointed out that regardless of fruit color, the mean fresh fruit mass is around 122 g; while Arriaga-Ruiz et al. [20] mentioned a range of fresh fruit mass between 49 and 100 g and equatorial diameters between 5 and 7 cm. The latter authors pointed out that the flesh mass and total soluble solids ranged between 65% and 75% and 11% and 14%, respectively. They also stated some ranges of pH, malic acid, proteins, and vitamins in both peel and flesh of pitayas; meanwhile, fresh and peel colors were visually determined. Another aspect, not less important, is the presence of numerous seeds in the pitayas’ flesh [2]. For instance, like in prickle pear [22] and in table grapes [23], the absence of seeds in pitaya flesh is a required attribute not only by consumers but also for by-product elaboration such as pitaya juice powders [24]. Hence, during juice preparation, seeds are removed and then some functional properties determined [24,25]. The same occurs with pitahaya juice preparation (*Hylocereus* spp.) [26,27,28]. Nerveless, seeds make great contributions to nutritional and functional properties for food elaboration [29]. Therefore, this study tested the hypothesis that juice powders with or without seeds of pitayas do not modify some phytochemical and functional attributes. Some physicochemical traits of pitayas were also assessed to add knowledge to those *S. queretaroensis* pitayas grown in other agro-ecological places [20,21].

## 2. Results

### 2.1. Quality and Color Attributes

**Figure 1:**
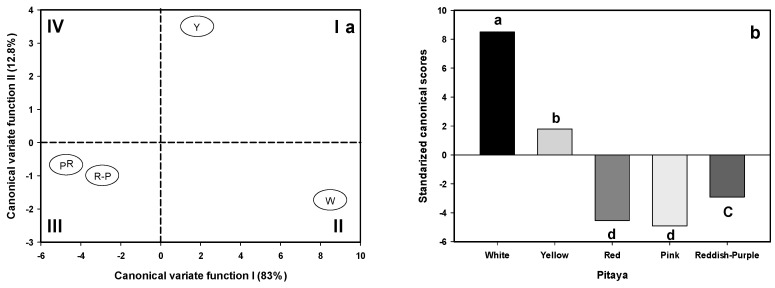
Standardized canonical scores of the first two canonical variate functions of some quality attributes of ‘White’ (W), ‘Yellow’ (Y), ‘Red’ (R), ‘Pink’ (P), and ‘Reddish-Purple’ (R-P) pitayas (*S. queretaroensis*) (**a**) and mean standardized canonical scores of the first canonical variate function (**b**). Different lowercase among bars indicates significant differences by minimum significant difference (MSD = 1.615) of Scheffe’s test at *p* < 0.0001.

‘White’ pitaya had the lowest mean values of fruit dimensions, while its peel and flesh color attributes were the greatest. The peel brightness (PL*) of the ‘White’ pitaya was lighter compared to the group of red and yellow ones. The peel chromaticity (PC*) of the ‘White’ pitaya was less dark than the other pitaya peels. The ‘White’ and ‘Yellow’ pitaya peels, in terms of Hue angle (H*), tended to be greener, while the peels of ‘Red’, ‘Pink’, and ‘Reddish-Purple’ pitayas were reddish. Flesh color attributes followed similar color patterns to those observed on the peels. ‘Pink’ pitaya had the greater total soluble solids concentration (TSSC), while the lowest value was observed in the ‘White’ pitaya and the remainder of the pitayas were placed in-between (Table 1). However, in order to have a multivariate overview of the differences among pitaya groups, after multivariate normal testing (χ^2^ = 0.851), a canonical variate analysis (CVA) was applied to the raw fruit physicochemical data. So, based on Wilks’ Lambdas’ test (*p* < 0.0001; Table 2), the first two canonical variates functions (CVF I and CVF II) accounted for separation among pitaya groups along with the large eigenvalues, explained variance (95.8%), and squared canonical correlation values (Table 2). Based on the correlation between the original variables (R) and the CVF I, there is a contrast suggested between fruit dimensions (except for peel mass, PM) and TSSC and peel and flesh color attributes (except for flesh chromaticity, FC*). In contrast, the CVF II was dominated positively by PM and FC* (Table 2). Consequently, the positioning of the ‘White’ pitaya (Quadrant II, Figure 1a) seems to be loaded towards higher scores of both peel and flesh color attributes and lower MP, TSSC, and FC* scores compared with those values observed in the ‘Yellow’ pitaya (Quadrant I; Figure 1a). In contrast, reddish pitayas were placed in Quadrant III due to low scores of both peel and pulp color attributes rather than their fruit dimension scores (Quadrant III; Figure 1a). The last description was confirmed and summarized by submitting the standardized canonical scores of the CVF I to analysis of variance and *post hoc* minimum significant difference of the Fisher’s test at *p* ≤ 0.05 (Figure 1b).

### 2.2. Phytochemical Characterization

#### 2.2.1. Non-Digestible Carbohydrates

There was significant interaction between the levels ×pitayas (PF, ‘White’, W; ‘Yellow’, Y; ‘Red’ (R); ‘Pink’ (P), and ‘Reddish-Purple’, R-P) and juice powder (JP) levels (JPS or JPWS) for total dietary fiber (TDF), insoluble dietary fiber (DF), and resistant starch (RS), except for soluble dietary fiber (SDF). In accordance with the main effect of ×pitayas (PF), RP and P pitayas had the highest mean values of TDF, respectively, while the mean effect of JP pointed out that the highest TDF mean value was found in JPS (Table 3). In contrast, overall, the interaction between PF × JP produced greater mean TDF, IDF, and RS values in all pitayas with JPS compared with those pitayas that were analyzed without seed. Nonetheless, the interactions Y × JPS, R × JPS, and W × JPS had the highest mean values of TDF, IDF, and RS, respectively (Table 3).

#### 2.2.2. Secondary Metabolism Phytochemicals

The concentration of phenolic compounds was differentially modified by the interaction PF × JP. While ‘Yellow’ × JPWS had the highest mean of total phenols, the highest mean values of total flavonoids, total anthocyanins, and total proanthocyanidins were observed in the ‘Pink’ × JPWS. In contrast, the mean values of total carotenoids and total saponins were greater in Y × JPWS and P × JPS, respectively, than the remaining interactions (Table 4). The concentrations of betaxanthins (BX) and indicaxanthins (IX) were also differentially modified by the interaction PF × JP. The interactions ‘Yellow’ × JPWS and ‘White’ × JPWS had the highest and no measurable values of BX and IX, respectively. The BX and IX mean concentrations were not detected in the ‘White’ × JPS and ‘Reddish-Purple’ × JPWS interactions. Similarly, the interactions ‘Yellow’ × JPS, ‘White’ × JPWS, and ‘Yellow’ × JPWS did not detect values of IX either (Table 4).

### 2.3. Functional Properties

On average, the ‘Reddish-Purple’ × JPS and ‘Pink’ × JPS interactions had the greatest antioxidant capacity, in terms of ABTS and DPPH, respectively. The lowest mean values of ABTS and DPPH were observed in the ‘Yellow’ ×JPS and ‘Reddish-Purple’ × JPWS interactions, respectively. The strongest pancreatic lipase (PL) inhibition was produced by the ‘Red’ × JPWS interaction, while ‘Pink’ × JPS had the weaker PL inhibition (Table 5).

## 3. Discussion

### 3.1. Quality and Color Attributes

**Figure 2:**
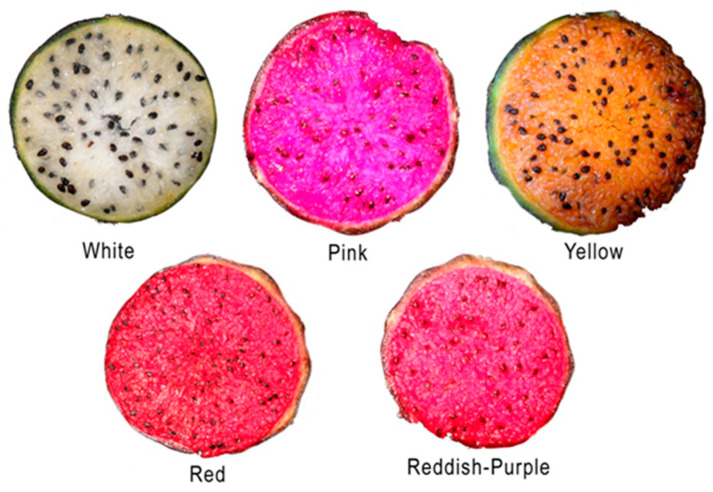
Transversal photographs of ‘White’, ‘Yellow’, ‘Red’, ‘Pink’, and ‘Reddish- Purple’ pitayas (*S. queretaroensis*).

In this study, pitayas’ dimensions and total soluble solids concentration (TSSC) were found within the ranges given by Arriaga-Ruiz et al. [20] (Table 1). However, these values differ from those reported by Pimienta-Barrios and Tomas [21]. Discrepancies may be associated with the location sampling and fruit ripening stage. While Arriaga-Ruiz et al. [20] collected their fruit in Santa Rosa, Juchipila Canyon at a distance of 37 km approximately from our fruit collection location, Pimienta-Barrios and Tomas [21] collected their fruit in the Sayula Basin approximately 275 km away from our study location. On the other hand, the lower values of TSSC determined by Pimienta-Barrios and Tomas [21] suggest that their fruit was less mature than here because our fruit was collected at the ready-to-eat stage, presumably similar to that collected by Arriaga-Ruiz et al. [20]. In fact, in accordance with the first canonical variate function (Figure 2), rather than the fruit dimensions attributes, the differences among these pitaya fruit were established by the peel and flesh colors, similar to those evaluated for *S. pruinosus* and *S. stellatus* [18].

### 3.2. Phytochemical Characterization

#### 3.2.1. Non-Digestible Carbohydrates

The evaluation of non-digestible carbohydrates has become an important aspect of the study of functional foods. The dietary fiber is known as the edible parts of plants that are resistant to digestion, and its ingestion produces many beneficial effects such as the prevention of obesity, diabetes, and cardiovascular diseases [30]. Interestingly, pigmented fruits often have a higher concentration of dietary fiber; according to Padayachee et al. [31], fruits and vegetables are good sources of dietary fiber in the form of the plant cell wall. Although the enzymes of pigment metabolism, such as polyphenols, are located in the endoplasmic reticulum, the main accumulation sites of these pigments are the cell walls [32]; this could explain why RP and P had the highest TDF content (Table 3). Overall, the concentration of dietary fiber was higher in JPS. This is because seeds contain fibrous structures to provide mechanical reinforcement by thickening secondary cell walls to protect the embryo and its resources (endosperm, perisperm) against physical, chemical, and biological damage [33]. Resistant starch is a type of starch that resists digestion in the small intestine and behaves more like dietary fiber [34]; however, the relationship between fruit color and resistant starch content is not straightforward.

#### 3.2.2. Secondary Metabolism Phytochemicals

The bioactive compounds, including pigments, are responsible for giving the smell, flavor, and color of agricultural commodities [35]. Nowadays, phenolic compounds are one of the most studied classes of bioactive pigments; these molecules have well-reported health benefits [36]. However, distribution of these compounds throughout different plant components have been reported [37]. Total phenols and flavonoids were higher in all JPS samples, in comparison to its corresponding fruit-colored JPWS (Table 4). This could be explained by the interaction of polyphenols and dietary fiber through covalent bonds, which allow the linkage of phenolics to cell wall structural components, ether bonds between lignins and hydroxyl groups of the phenolic ring, or esters between their carboxyl group and alcohol groups of polysaccharides and proteins [38].

Carotenoids, alongside anthocyanins and betalains, are the primary natural pigments used in the food industry because of their positive biological effects, especially in reducing the risk of some chronic diseases; these compounds provide orange, yellow, and red colors, due to their chromophores, which consist mainly of a chain of conjugated double bonds [10]. This agrees with the current results, since both ‘Yellow’ (JPS and JPWS) and JPWS of ‘Pink’ pitayas had the highest carotenoid concentration (Table 4).

Saponins are specialized plant terpenoids derived from the mevalonic acid pathway, which show hypocholesterolemic, immunoadjuvant, and anti-inflammatory activities, among others [39]. Saponins are also relevant products in the food industry. The capacity to interact with several membrane components and access these in various cell compart-ments as a result of their amphipathic properties seems to be at the center of triterpene saponins’ mechanism of action [40]. However, to the best of our knowledge, the relation-ship between fruit color and the concentration of saponin has not been investigated enough. Although results provide evidence of an interaction effect among fruits’ color and whether samples were analyzed with or without seeds, overall JPS had the highest saponin content (Table 4). In many cases, the saponin content in fruit seeds is relatively low compared to other parts of the plant, such as the leaves or roots. Nonetheless, the current results suggest that seeds provide a portion of the total saponins present in the whole fruit.

Betalains can be found in combination with polyphenols in pitayas, particularly hy-droxycinnamoyl derivatives, flavanols, and flavanones [25]. Nonetheless, compared to other pigments, such as anthocyanins and carotenoids, betalains have superior qualities, which is promising for their application in the industry; these include high water solubility, high stability, and color variability based on pH conditions, as well as the attractive characteristic of being odorless and tasteless [5]. According to Morales et al. [3], the range of colors from cacti betalains ranges from yellow to violet, more specifically, betaxanthins are yellow pigments from the conjugation of betalamic acid with the amino group of amino acid, which agrees with the current results (Table 4).

### 3.3. Functional Properties

*Stenocereus* fruit exhibits high antioxidant potential due to the presence of substances with the capacity to give up an electron or a hydrogen atom to scavenge oxidizing compounds, such as ascorbic acid, betalains, and different phenolic compounds ‘Reddish-Purple’ and ‘Pink’ fruits exhibited the highest antioxidant capacity evaluated by the inhibition percentage of ABTS and DPPH, respectively (Table 5). Both fruit colors had the highest anthocyanins concentration; according to Ali et al. [41] anthocyanins are a class of flavonoid responsible for red, blue, and purple colors in the plant kingdom, and the most common are those with the 3-glycoside structure. The number and position of hydroxyl and methoxy substituents, as electron donating groups, were found to have a great impact. Interestingly, the highest capacity to scavenge both, ABTS and DPPH radicals, were obtained by JPS, meaning that the antioxidant capacity is not only dependent on the color of the fruit per se, but also by the phytochemicals provided by other constituents of the fruit, such as seed, flesh, or peels. For instance, Herrera et al. [42] evaluated *Opuntia ficus-indica* peels as an outstanding source of phenolic compounds with a high capacity to scavenge ABTS and DPPH.

Evaluating the effect of a food on in vitro inhibition of digestive enzymes like the pancreatic lipase is a preliminary approach to determining its functional potential. This enzyme is capable of hydrolyzing up to 50–70% of total dietary fats that are absorbed, related to higher triglycerides intestinal absorption. Therefore, inhibiting the enzyme has been proposed as a target to dyslipidemia and atherosclerosis management, since the inhibition of hydrolyzing fats could be a strategy to avoid lipid metabolism and storage, reducing weight gain, and inflammation [42,43,44]. For this functional property, the highest capacity for inhibiting pancreatic lipase was observed by JPWS. This might be related to their concentration of total phenols and flavonoids, as JPWS exhibit an increase in these compounds (Table 5). For instance, total phenolics and flavonoids have been previously linked as efficient phytochemicals in enhancing lipase inhibition [42].

These findings highlight the importance of characterizing pitaya juices, with or without seeds. It is not new that the food industry has a greater focus on working with natural pigments; as mentioned before, betalains have been identified as pigments with superior characteristics, in comparison with anthocyanins and carotenoids, two of them being that they are odorless and tasteless. In regard the cosmetic industry, some brands have exploded into the market, using natural pigments such as flavonoids and anthocyanins, the latter being higher in some cases in JPS. Moreover, beneficial effects have been reported for dietary fiber; hence, the necessity of exploring new raw materials, with a balanced composition of fiber fractions and containing a high amount of associated bioactive compounds has been recognized. The ingestion of insoluble fiber from fruits and vegetables can produce a significant decrease in the plasmatic concentration of cholesterol, which implies a decrease in the risk of suffering cardiovascular disease [30]. Additionally, JPS exhibited better antioxidant capacity.

Contrary to the above, although JPWS had a higher concentration of betalains and some phenolic compounds, the current results demonstrate that JPS upholds the higher concentration of non-digestible carbohydrates. As for lipase inhibition, current pharmacological treatments for dyslipidemia, like statins, fibrate, niacin, and resins have led to adverse effects [45]. For this reason, exploring other resources may add to the search of novel pharmacological ingredients.

## 4. Materials and Methods

### 4.1. Study Area

The “Cañones” region of Zacatecas, México, has an extensive pitayo wild population between latitude 21° 54′ N, longitude 103° 14′ W, elevation 2041 m, and latitude 21° 13′ N, longitude 102° 45′ W, elevation 1739 m. The climate of this area is BS1 (h’)w, or warm semiarid with minimum, mean, and maximum annual temperatures of 9.8 °C, 19.7 °C, and 29.6 °C, respectively. Rainfall is between 500 and 582 mm per year, of which 75% occurs between July and September. The soil is characterized by an alkaline pH (8.2), with inorganic nitrogen and organic matter of 28.9 ppm and 1.1%, respectively. Potassium and calcium content is high, with values over 2300 and 5800 ppm, respectively. The pitayo shares this ecological system with other endemic species such as the cactus pear (*Opuntia* spp.), native grasses, tepame (*Acacia pennatula*), garanbullo (*Myrtillocactus geometrizans*), huizache (*Vachellia farnesiana*), white mezquite (*Prosopis laevigata*), Mexican kidneywood (*Eysenhardita polystachya*), pochote (*Ceiba aesculifolia*), granjel (*Bunchosia palmeri*), and maguey (*Agave* spp.), among others. Pitayo genotypes produce fruit with white, yellow, red, pink, and reddish-purple flesh and the local people call them pitaya with their colors (e.g., white pitaya, red pitaya, etc., Figure 2).

### 4.2. Fruit Sampling

Pitayo fruit is highly perishable, and, therefore, the fruit was collected at a ready-to-eat stage early in the morning on 25 May 2018 from local growers previously contacted in Jalpa, Zacatecas. The fruit was placed into potable coolers and transported immediately for processing to the Campo Experimental Zacatecas (latitude 22°54′ N, 102°39′ W, elevation 2197 m) of the Instituto Nacional de Investigaciones Forestales, Agrícolas y Pecuarias (INIFAP) in Mexico located at distance of 171 km to the north-east (approximately two and half hours).

### 4.3. Physicochemical Characterization

Five lots, each of ten fruits of white, yellow, red, pink, and reddish-purple fruits (Figure 2) were collected. The polar and equatorial diameters were recorded individually with a digital caliper (Model CD-6, CS, Mitutoyo Co., Tokyo, Japan). The mass of each fruit was recorded with a precision balance (VE-303, Velab, Mission Viejo, CA, USA). Each fruit was separated into peel and pulp, and their masses were weighed separately with the same scale, and the edible portion was estimated. Peel and flesh color, in terms of lightness, chromaticity, and hue angle, were measured with a spectrophotometer (Model-SP64, X-Rite Inc., Grandville, MI, USA). Some juice drops were mixed and the total soluble solids concentration was measured with a digital refractometer with automatic temperature compensation (model PR-32α, Atago, Co., Ltd., Tokyo, Japan).

### 4.4. Sample Preparation

The juice of ten pitayas of each color was stored at −70 °C before lyophilization (model 7754041, LABCONCO^®^, Kansas City, MO, USA). This process lasted seven days, which was mandatory to obtain dried samples with less than 0.5% moisture content. Moisture content was determined and no significant differences were observed among samples. Afterwards, samples were separated in two groups of five fruits each. The first group of each color was processed to obtain pitaya juice powders with seeds (JPS) and the rest were sifted using an ultrafine nylon sifter to separate the dry juice from the seeds (JWS). All samples were stored in sealed bags under fresh and dark conditions until analysis.

### 4.5. Phytochemical Characterization

#### 4.5.1. Non-Digestible Carbohydrates

The content of total dietary fiber (TDF) was determined using the total dietary fiber assay kit (Sigma-Aldrich, St. Louis, MO, USA). The content of resistant starch was determined from the insoluble fraction of the TDF as described previously [46]. The final glucose concentration was analyzed using a GOD-PAP kit (Randox Laboratories Ltd., Gortnagallon, Crumlin, UK), and a glucose standard solution (0–0.98 mg/dL) was used as control. The resistant starch was calculated as glucose (mg) × 0.9.

#### 4.5.2. Phenolic Compounds

Extracts were obtained from a 1 g sample mixed with 10 mL acetone/water/acetic acid (70:29.5:0.5, *v*/*v*/*v*, respectively) as described by Xu and Chang [47]. The total phenolic content was determined by the Folin–Ciocalteu assay [48]. The concentration was expressed as mg gallic acid equivalents per g dry sample (mg GAE/g). Proanthocyanidins were determined with the vanillin-HCl method as previously described [49], and total flavonoids were quantified by the AlCl3 colorimetric assay [50]. The concentration of condensed tannins and total flavonoids was expressed as mg (+)-catechin equivalents per g dry sample (mg CAE/g). Anthocyanin content was determined using an ethanolic extract as described by Abdel-Aal et al. [51] and expressed as mg cyanidin-3-glucoside equivalents per 100 g dry sample using a coefficient of 26,900 L/cm mol. and a molecular weight of 449.2 g/mol.

#### 4.5.3. Total Carotenoids

The carotenoid extraction from 5 mg of freeze-dried prickly pear peels was performed by adding 1 mL of ACN:MeOH:THF (50:45:5, by volume) and by shaking at 150 rpm for 2 h. The samples were centrifuged at 4000 rpm for 5 min at −5 °C and the supernatant was recovered. The total carotenoid content was determined by measuring the absorbance at 450 nm. The samples extracted with the solvent mixture described above and the standard diluted in the same solution were analyzed. The total carotenoid content was expressed on a β-carotene basis [52].

#### 4.5.4. Total Saponins

The total saponins were extracted from 0.5 g of dried samples with 4 mL of hexane over 6 h, and the mixture was centrifuged at 19,000× *g* for 5 min. The hexanic phase was recovered, evaporated to dryness, and dissolved in 200 μL of acetonitrile [53]. For the total saponins quantification, 50 μL of each sample was mixed with 500 μL of vanillin (8% *w*/*v*) and 5 mL of sulfuric acid (72% *v*/*v*). The mixture was incubated at 60 °C for 10 min, cooled in an ice water bath, and the absorbance was measured at 538 nm. The results were expressed as micrograms of oleanolic acid equivalents per milliliter (µg OAE g-1).

#### 4.5.5. Betalains

The sample was mixed with methanol (1:5 (*w*/*v*, respectively)) and homogenized by magnetic stirring. The mixture was filtered with a nylon filter (0.45 µm). The absorbance of methanolic extracts was measured by using a microplate reader (Multiskcan Go, Thermo Scientific, Oy Ratastie 2, Finland). The quantity of pigment from the pitayas’ flesh extract was determined by spectrophotometry according to Lambert Beer’s law: A = log (I10/I) = ε × L × c, after the absorbance measurement at a languid wave related to the maximum absorption of betaxanthin (482 nm) and indicaxanthin (532 nm). The molar extinction coefficients were 62,000, L/mol cm for betaxanthin, and 48,000 L/mol cm for indicaxanthin [54]. The data were expressed as mg of pigment per kg of fruit peel [55].

### 4.6. Functional Characterization

#### 4.6.1. Antioxidant Capacity

The antioxidant capacity was assessed as the ability of methanolic peel extracts to scavenge the stable radical 1,1-diphenil-2-picrylhydrazyl (DPPH) as described [56]. The 2,2-azinobis-3-ethylbenzothiazoline-6-sulfonic acid (ABTS) free radical scavenging capacity of samples was evaluated as described by Machado et al. [57] and expressed as inhibitory concentration of 50% (IC50), the concentration required to scavenge 50% of the radicals.

#### 4.6.2. Pancreatic Lipase Inhibition

To assess the inhibition of pancreatic lipase, porcine pancreatic lipase type II was dissolved in distilled water (10 mg/mL, *w*/*v*). The supernatant was recovered after centrifugation at 13,000× *g* for 5 min. The buffer used for the determination was 100 mM Tris buffer (pH 8.2) and the substrate was 0.08% (*w*/*v*) p-nitrophenyl laurate (PNP) dissolved in 5 mM sodium acetate (pH 5.0) with 1% Triton X-100. The solution was heated for 1 min in boiling water, then well mixed and cooled to room temperature. For the determination, each tube contained 400 μL assay buffer, 450 μL substrate solution, 150 μL pancreatic lipase, and 450 μL of flesh extract. The samples were incubated at 37 °C for 2 h, centrifuged at 13,000× *g* for 2.5 min, and the absorbance was read at 400 nm as described by McDougall et al. [58].

### 4.7. Statistical Analysis

Fruit physicochemical attributes were analyzed first with descriptive statistics of fruit colors. Then, after multivariate normality testing, all the information was arranged to be analyzed collectively by using the canonical variate method. The differences among pitayo fruits (PF), if any, were analyzed with a completely random model using the canonical scores of the first canonical variate function. Differences among PF were separated with the *post hoc* minimum significant difference of the Scheffe’s test at *p* ≤ 0.05. Phytochemical and functional data were analyzed with a completely random model with a factorial arrangement in the treatments (PF × JP). Differences due to interaction effects were separated by the *post hoc* minimum significant difference of the Fisher’s test at *p* ≤ 0.05. The information was analyzed with the statistical analysis system (SAS Institute ver. 9.4, 2002–2010, Cary, NC, USA).

## 5. Conclusions

In summary, this study suggested that differences among pitayas were related to peel and flesh color attributes rather than fruit dimensions. The results suggest rejecting our hypothesis because the absence of seeds in all pitaya juice diminished non-digestible carbohydrate compounds, except for soluble dietary fiber; while enhanced phenolic compounds were in all pitaya juices, the total saponins also increased in the interaction ‘Pink’ × juice powder with seeds (JPS). The interaction of ‘Yellow’ × juice powders without seeds (JPWS) had the highest content of betaxanthins, but the highest indicaxanthins content was observed in the interactions ‘Pink’ × JPS and ‘Pink’ × JPWS. In contrast, the antioxidant capacity, in terms of ABTS and DPPH scavenging, was the highest in the interactions ‘Reddish-Purple’ × JPS and ‘Pink’ × JPS, respectively. The lowest and greater pancreatic lipase inhibition was observed with the interaction ‘Pink’ × JPS and ‘Red’ × JPWS, respectively. Therefore, except for total phenols, the flesh and seeds of these fruits have more benefits for human health when consumed fresh or as a by-product. However, the latter needs to be explored further.

## Figures and Tables

**Table 1:** Mean values (±95% confidence intervals) of some quality and color attributes ^1^ of pitayas (*S. queretaroensis*).

Pitayas	ED (mm)	PD (mm)	FM (g)	PM (g)	FshM (g)	EP (%)	Peel Color Attributes			Flesh Color Attributes			TSSC
							L*	C*	H*	L*	C*	H*	
White	47.1 ± 2.6	49.6 ± 2.7	58.9 ± 7.5	20.0 ± 2.9	38.9 ± 2.9	65.8 ± 4.1	38.1 ± 3.6	18.5 ± 6.0	98.0 ± 6.0	59.2 ± 2.4	11.5 ± 1.5	91.9 ± 1.4	10.9 ± 1.1
Yellow	53.2 ± 4.1	54.8 ± 4.0	85.5 ± 10.6	24.8 ± 4.0	60.7 ± 8.6	70.8 ± 8 4.0	34.7 ± 1.9	12.7 ± 2.5	86.4 ± 10.2	49.9 ± 2.7	37.3 ± 4.0	56.7 ± 6.1	12.5 ± 1.5
Red	50.1 ± 2.0	55.2 ± 3.4	72.5 ± 9.6	22.3 ± 3.5	50.9 ± 9.5	69.6 ± 5.2	28.9 ± 0.7	7.9 ± 1.9	29.8 ± 3.2	36.9 ± 2.4	27.2 ± 2.7	19.9 ± 1.5	12.5 ± 1.5
Pink	51.0 ± 7.8	53.5 ± 7.7	77.2 ± 26.0	21.1 ± 7.3	55.1 ± 23.0	70.5 ± 11.6	30.8 ± 3.4	10.1 ± 4.2	33.5 ± 26.9	39.0 ± 7.9	25.9 ± 10.6	14.6 ± 8.6	13.0 ± 3.0
Reddish-Purple	53.3 ± 3.7	55.1 ± 3.8	81.7 ± 17.4	20.8 ± 3.5	61.4 ± 18.7	73.1 ± 7.8	30.3 ± 1.4	8.9 ± 1.8	38.3 ± 7.2	36.8 ± 2.4	25.1 ± 5.9	26.1 ± 4.4	12.1 ± 0.8

^1^ Equatorial diameter = ED; Polar diameter = PD; Fruit mass = FM; Peel mass = PM; Flesh mass = FshM; Edible portion = EP; Brightness = L*; Chromaticity = C*; Hue angle = H*; Total soluble solids concentration (%) = TSSC.

**Table 2:** Correlation coefficients (r) between the first two canonical variates functions (CVF1 and CVF2) and 13 fruit quality attributes of five pitayas (*S. queretaroensis*).

Fruit Quality Attributes	CVF1	CVF2
	r	r
Fruit mass (g)	−0.30	0.39
Peel mass (g)	−0.05	0.38
Flesh mass (g)	−0.30	0.30
Edible portion (%)	−0.24	0.11
Polar diameter (cm)	−0.36	0.21
Equatorial diameter (cm)	−0.28	0.31
Peel lightness	0.78	0.11
Peel chromaticity	0.68	−0.04
Peel Hue angle	0.90	0.34
Flesh lightness	0.92	0.09
Flesh chromaticity	−0.43	0.80
Flesh Hue angle	0.99	0.05
Soluble solids (%)	−0.31	0.21
Eigenvalue	28.2	4.3
Variance explained (%)	83.0	12.8
Squared canonical correlation	0.98	0.87

**Table 3:** Main and interaction effects of pitayo fruit (PF) and juice powders (JP) with (JPS) or without seeds (JPWS) on non-digestible carbohydrates (g/100 g).

Source of Variation	Non-Digestible Carbohydrates (g/100 g)			
	TDF *	SDF	IDF	RS
Main effects				
Pitayo fruit (PF)				
‘White’ (W)	3.8 ± 0.9 a ^1^	2.3 ± 0.4 a	1.6 ± 0.6 c	0.7 ± 0.3 a
‘Yellow’ (Y)	4.1 ± 1.3 a	1.9 ± 0.6 ab	2.3 ± 0.8 bc	0.5 ± 0.2 a
‘Red’ (R)	3.4 ± 1.2 a	1.0 ± 0.3 cd	2.4 ± 1.0 bc	0.6 ± 0.3 a
‘Pink’ (P)	4.4 ± 0.7 a	1.5 ± 0.3 bc	2.9 ± 0.5 b	0.6 ± 0.2 a
‘Reddish-Purple’ (RP)	4.6 ± 0.2 a	0.6 ± 0.3 d	3.8 ± 0.3 a	0.5 ± 0.1 a
Least significant difference	1.2	0.7	0.9	0.3
Significance (*p* > F)	0.232	0.001	0.001	0.339
Juice Powders (JS)				
Juice powder with seeds (JPS)	5.8 ± 0.3 a	2.1 ± 0.3 a	3.7 ± 0.2 a	1.0 ± 0.1 a
Juice powder without seeds (JPWS)	2.3 ± 0.3 b	0.8 ± 0.1 b	1.5 ± 0.4 b	0.2 ± 0.03 b
Least significant difference	0.7	0.5	0.5	0.2
Significance (*p* > F)	<0.0001	<0.0001	<0.0001	<0.0001
InteraciInteraction effects (PF × JP)				
W × JPS	5.9 ± 0.2 ab	3.1 ± 0.3 a	2.8 ± 0.4 bc	1.8 ± 0.4 a
Y × JPS	6.6 ± 2.4 a	2.9 ± 1.6 a	3.7 ± 1.6 ab	0.9 ± 0.3 bc
R × JPS	5.9 ± 1.1 ab	1.4 ± 0.5 a	4.5 ± 0.5 a	1.2 ± 0.2 ab
P × JPS	6.0 ± 1.0 ab	2.1 ± 0.3 a	3.9 ± 1.0 ab	0.9 ± 0.3 bc
RP × JPS	4.8 ± 0.4 b	1.3 ± 0.4 a	3.6 ± 0.3 ab	0.6 ± 0.5 c
W × JPWS	1.8 ± 0.6 de	1.5 ± 0.4 a	0.3 ± 0.2 e	0.1 ± 0.03 e
Y × JPWS	1.7 ± 0.5 de	0.9 ± 0.1 a	0.9 ± 0.4 de	0.1 ± 0.04 e
R × JPWS	0.9 ± 0.6 e	0.6 ± 0.4 a	0.3 ± 0.2 e	0.1 ± 0.01 e
P × JPWS	2.9 ± 0.2 cd	1.0 ± 0.2 a	1.9 ± 0.1 cd	0.3 ± 0.02 de
RP × JPWS	4.4 ± 0.6 bc	0.04 ± 0.02 a	4.0 ± 1.0 ab	0.3 ± 0.1 de
Least significant difference	1.7	2.6	1.2	0.4
Significance (*p* > F)	0.004	0.512	0.0004	0.008
Coefficient of variation (%)	23.9	41.1	27.8	40.2

* Total dietary fiber (TDF), soluble dietary fiber (SDF), insoluble dietary fiber (DF), and resistant starch (RS). ^1^ Values are the means ± standard deviation. Different low-cases within columns indicate significant difference by Fisher’s at *p* < 0.05.

**Table 4:** Interaction effects of pitayo fruits and juice powders with (JPS) or without seeds (JPWS) on phytochemical compounds.

Source of Variation	TP * (mg EGA/g)	TF (mg EGA/g)	TA (mg EC3G/g)	TPA (mg ECA/g)	TC (mg EβC/g)	TS (mg EAO/g)	BX	IX
							(mg/g)	(mg/g)
Interaction effects								
‘White’ × JPS	1.8 ± 0.1 h ^1^	0.8 ± 0.1 d	0.03 ± 0.0 e	4.3 ± 0.3 d	10.3 ± 0.9 f	0.3 ± 0.2 de	0.0 d	0.0 c
‘Yellow’ × JPS	11.3 ± 0.7 d	0.8 ± 0.1 d	0.07 ± 0.0 e	3.4 ± 0.3 e	25.8 ± 1.5 b	0.4 ± 0.0 abc	0.71 ± 0.01 c	0.0 c
‘Red’ × JPS	7.0 ± 0.7 f	0.7 ± 0.04 e	0.6 ± 0.1 c	7.3 ± 0.5 c	22.8 ± 2.3 d	0.5 ± 0.0 ab	0.03 ± 0.006 d	0.01 ± 0.0 b
‘Pink’ × JPS	5.8 ± 0.6 g	0.9 ± 0.09 cd	1.2 ± 0.02 a	16.8 ± 0.5 b	14.9 ± 0.7 e	0.5 ± 0.1 a	0.02 ± 0.01 d	0.02 ± 0.0 a
‘Reddish-Purple’ × JPS	10.3 ± 1.0 e	0.6 ± 0.04 e	1.2 ± 0.02 a	4.5 ± 0.4 d	23.5 ± 1.1 cd	0.3 ± 0.0 cde	0.02 ± 0.01 d	0.01 b
‘White’ × JPWS	2.2 ± 0.1 h	1.0 ± 0.03 bc	0.03 ± 0.0 e	7.0 0.2 c	10.8 ± 0.5 f	0.4 ± 0.1 bcd	0.01 ± 0.01 d	0.0 c
‘Yellow’ × JPWS	17.8 ± 0.05 a	0.9 ± 0.1 d	0.06 ± 0.0 e	4.9 ± 0.06 d	31.4 ± 2.0 a	0.2 ± 0.0 e	2.20 ± 0.35 a	0.0 c
‘Red’ × JPWS	16.0 ± 0.5 b	0.6 ± 0.05 e	0.9 ± 0.08 b	5.0 ± 0.3 d	25.6 ± 0.9 bc	0.4 ± 0.0 bcd	1.28 ± 0.06 b	0.013 ± 0.012 b
‘Pink’ × JPWS	10.6 ± 0.3 ed	1.2 ± 0.05 a	1.2 ± 0.07 a	17.9 ± 0.7 a	27.0 ± 0.7 b	0.2 ± 0.0 e	0.02 ± 0.00 d	0.02 ± 0.0 a
‘Reddish-Purple’ × JPWS	14.6 ± 0.2 c	1.2 ± 0.2 ab	0.3 ± 0.04 d	5.0 ± 0.9 d	13.4 ± 1.3 e	0.4 ± 0.2 bcd	0.0	0.0 c
Least significant difference	0.9	0.13	0.08	0.8	2.2	0.14	0.2	0.006
Significance (*p* > F)	<0.0001	<0.0001	<0.0001	<0.0001	<0.0001	0.001	<0.0001	0.05
Coefficient of variation (%)	5.5	9.1	8.6	6.1	6.4	25.6	26.4	49.8

* Total phenols (TP), total flavonoids (TF), total anthocyanins (TA), total proanthocyanidins (TPA), total carotenoids (TC), total saponins (TS), betaxanthins (BX), and indicaxanthin (IX). ^1^ Values are the means ± standard deviation. Different low-cases within columns indicate significant differences using Fisher’s test at *p* < 0.05.

**Table 5:** Interaction effects of pitayo fruit and juice powders with (JPS) or without seeds (JPWS) on functional properties (%).

Source of Variation	ABTS	DPPH	PL
Interaction effects			
‘White’ × JPS	14.6 ± 1.2 e ^1^	56.1 ± 2.2 e	35.8 ± 0.2 d
‘Yellow’ × JPS	11.3 ± 3.2 e	84.2 ± 0.2 c	60.9 ± 2.1 b
‘Red’ × JPS	22.5 ± 1.2 b	78.2 ± 2.4 d	46.9 ± 8.0 c
‘Pink’ × JPS	18.4 ± 0.8 c	89.1 ± 0.2 a	28.5 ± 3.7 e
‘Reddish-Purple’ × JPS	34.0 ± 0.1 a	87.6 0.6 ab	39.5 ± 6.2 d
‘White’ × JPWS	6.1 ± 0.3 e	22.7 ± 1.1 f	47.4 ± 1.4 c
‘Yellow’ × JPWS	19.2 ± 2.5 bc	23.5 ± 0.8 f	65.0 ± 1.7 ab
‘Red’ × JPWS	12.6 ± 1.7 d	20.1 ± 0.4 g	67.4 ± 2.9 a
‘Pink’ × JPWS	14.6 ± 1.8 d	86.1 ± 0.8 bc	59.6 ± 1.4 b
‘Reddish-Purple’ × JPWS	22.7 0.3 b	20.1 ± 0.4 g	62.1 ± 0.8 ab
Least significant difference	3.6	2.6	6.3
Significance (*p* > F)	<0.0001	<0.0001	<0.0001
Coefficient of variation (%)	9.2	2.1	7.2

* ABTS and DPPH scavenging capacity and pancreatic lipase inhibition (PL). ^1^ Values are the means ± standard deviation Different low-cases within columns indicate significant difference using Fisher’s test at *p* < 0.05.

## Data Availability

Data will be made available on request.
